# Effect of a high-dose target-controlled naloxone infusion on pain and hyperalgesia in patients following groin hernia repair: study protocol for a randomized controlled trial

**DOI:** 10.1186/s13063-015-1021-6

**Published:** 2015-11-10

**Authors:** Manuel Pedro Pereira, Mads Utke Werner, Joergen Berg Dahl

**Affiliations:** Department of Anaesthesiology, Centre of Head and Orthopaedics, Rigshospitalet, Copenhagen University Hospitals, Copenhagen, Denmark; Multidisciplinary Pain Center, Neuroscience Center, Rigshospitalet, Copenhagen University Hospitals, Copenhagen, Denmark

**Keywords:** Central sensitization, Endogenous opioids, Humans, Latent sensitization, Naloxone, Open groin hernia repair, Pain, Randomized controlled trial, Secondary hyperalgesia, Target-controlled infusion

## Abstract

**Background:**

Central sensitization is modulated by the endogenous opioid system and plays a major role in the development and maintenance of pain. Recent animal studies performed following resolution of inflammatory pain showed reinstatement of tactile hypersensitivity induced by administration of a mu-opioid-antagonist, suggesting latent sensitization is mediated by endogenous opioids. In a recent crossover study in healthy volunteers, following resolution of a first-degree burn, 4 out of 12 volunteers developed large secondary areas of hyperalgesia areas after a naloxone infusion, while no volunteer developed significant secondary hyperalgesia after the placebo infusion. In order to consistently demonstrate latent sensitization in humans, a pain model inducing deep tissue inflammation, as used in animal studies, might be necessary. The aim of the present study is to examine whether a high-dose target-controlled naloxone infusion can reinstate pain and hyperalgesia following recovery from open groin hernia repair and thus consistently demonstrate opioid-mediated latent sensitization in humans.

**Methods/Design:**

Patients submitted to unilateral, primary, open groin hernia repair will be included in this randomized, placebo-controlled, double-blind, crossover study. The experimental days take place 6–8 weeks after surgery, time-points at which patients are expected to be almost pain- free. Prior to administration of naloxone or placebo, the primary outcome (a summated measure of pain: at rest, during transition from supine to standing position, and evoked by pressure algometry) and the secondary outcomes (secondary hyperalgesia/allodynia, pressure pain thresholds, assessed at the surgical site and at the mirror-site in the contralateral groin, and, opioid withdrawal symptoms) will be assessed. These assessments will be repeated at each step of the target-controlled infusion of placebo or naloxone at estimated median (95 % CI) plasma concentrations of 344 ng/ml (130;567), 1059 ng/ml (400;1752) and 3196 ng/ml (1205;5276).

**Discussion:**

We aim to demonstrate opioid-mediated latent sensitization in a post-surgical setting, using pain as a clinical relevant variable. Impairment of the protective endogenous opioid system may play an important role in the transition from acute to chronic pain. In order to sufficiently block the endogenous opioid system, a high-dose target-controlled naloxone-infusion is used, in accordance with recent findings in animal studies.

**Trial registration number:**

EUDRACT: 2015-000793-36 (Registration date: 16 February 2015)

Clinicaltrials.gov: NCT01992146 (Registration date: 12 December 2014)

## Background

Central sensitization is a condition in which the central nervous system is regulated into a state of high reactivity producing augmented responses. It plays a major role in the *development* and *maintenance* of pain [1, 2] and is modulated by the endogenous opioid system, which is known to be impaired or altered in various chronic pain conditions [2–4]. Mu-opioid-receptor (MOR)-antagonists can be used to block the endogenous opioid system and thereby to study the role of the endogenous opioids on central processing of pain.

Experimental studies in rodents showed a phenomenon of long-lasting vulnerability to noxious stimuli mediated by endogenous opioids, termed latent sensitization [5]. Following resolution of inflammatory pain, administration of MOR-antagonists led to *N*-methyl-*d*-aspartic acid (NMDA)-receptor dependent reinstatement of hypersensitivity at the injury site [6, 7]. Interestingly, naloxone-methiodine [6] and naltrexone-methobromide [7], two peripherally acting opioid antagonists not crossing the blood-brain barrier, did not change nociceptive thresholds following resolution of the inflammatory injury, confirming that latent sensitization is centrally mediated. Furthermore, intrathecal administration of pertussis toxin, disrupting Gα-subunit (Gα_i/o_-proteins), generated reinstatement of mechanical hypersensitivity, suggesting tonic activation of G-protein-coupled receptors [7]. Translational research is needed to study the phenomenon of latent sensitization and its modulation by the endogenous opioid system in humans.

Human experimental and clinical pain models [8, 9] can be used to study central sensitization. One feature of central sensitization is secondary hyperalgesia, where sensory stimulation of normal tissue adjacent to an injury is perceived as painful. Secondary hyperalgesia is thus characterized by expansion of receptive fields of dorsal horn neurons [1, 2]. The phenomenon of latent sensitization has not yet been robustly shown in humans. In a previous study in healthy volunteers, blockade of the endogenous opioid system by low-dose naloxone (0.021 mg/kg) did not significantly affect the magnitude of the areas of secondary hyperalgesia following resolution of a first-degree burn-injury [10]. However, following administration of high-dose naloxone (2 mg/kg), 4 out of 12 volunteers developed large secondary areas of hyperalgesia), while no volunteer developed significant secondary hyperalgesia during a placebo infusion [11]. These findings, though not as robustly shown as in rodents, suggest that latent sensitization, modulated by the endogenous opioid system, is present in humans.

The burn-injury model, applied in the human studies is a validated, tonic thermal injury model, which has been widely used in pain studies in healthy volunteers [12, 13]. This model induces, however, only superficial inflammatory damage, in contrast to the deep tissue inflammation induced by plantar incision [6] or by intra-plantar injection of complete Freund’s adjuvant (CFA) [7] used in animal studies. A more invasive pain model, inducing deep tissue inflammation, thus compatible with the clinical scenario, might be necessary to consistently generate latent sensitization in humans. We therefore opted for a post-surgical model in the present study. Open groin hernia repair (GHR) induces very likely a higher degree of nociception compared to the burn-injury model and is associated with long-lasting peripheral and central sensitization [14].

We hypothesize that a high-dose naloxone infusion will reinstate pain (resting pain, dynamic pain and pressure evoked pain) and hyperalgesia 6 to 8 weeks after primary unilateral, open GHR with mesh implantation. Although at this time interval patients are expected to be nearly pain-free, tissue injury induced by surgery has not yet completely resolved. The endogenous opioid system has a protective function and likely contributes to the pain-free state and to a more rapid regaining of function of the affected area [15–17]. Impairment of the endogenous opioid system may be an important pathophysiological mechanism in persistent pain, after surgery or trauma. By blocking the endogenous opioid system, using a target-controlled infusion (TCI) of high-dose naloxone, we expect to observe a reinstatement of pain evoked by the surgically induced tissue damage. Noteworthy, in the absence of an injury it is unlikely that naloxone causes pain, since studies in healthy volunteers using naloxone doses in the mg/kg range reported behavioral adverse effects as nausea or tiredness but, except for epigastralgia, pain has not been described [18–22].

The *primary aim* of this study is thus to examine the effect of a naloxone high-dose TCI (total dose: 3.25 mg/kg) on pain reinstatement after recovery from GHR. *Secondary aims* are to study the effect of naloxone on secondary hyperalgesia, allodynia and pressure pain thresholds (PPT), at the surgical site and at the mirror-site in the contralateral groin. Opioid withdrawal symptoms and psychometric variables: i.e., anxiety, depression and pain catastrophizing behavior, will also be assessed.

## Methods/Design

### Participants

Patients submitted to unilateral, primary open GHR by the Lichtenstein procedure will be recruited at the Department of Surgical Gastroenterology at Gentofte/Herlev Hospital. The investigator will provide participants with oral and written information about the study and its possible risks. Informed consent from each participant will be obtained. Participants will receive a compensation of EUR 20 (USD 27) per hour for their participation in the study. Inclusion and exclusion criteria are presented in Table 1.

**Table 1:** Inclusion and exclusion criteria

Inclusion criteria	Exclusion criteria
• Healthy male (ASA I–II)	• Participant does not understand Danish
• 18 ≤ age ≤ 65 years	• Participant is not cooperative
• Patients submitted to elective, unilateral, primary, open groin herniarepair 6–8 weeks prior to study start	• Participant had previous surgery in the inguinal region
• Open surgical procedure by the Lichtenstein method	• Pain at rest > 3 (NRS, 0–10)
• Signed informed consent	• Activity-related pain in the surgical field > 5 (NRS, 0–10)
• 18 kg/m^2^ < BMI < 30 kg/m^2^	• Nerve lesions in the assessment sites (for instance, after trauma, disc herniation, etc.)
• Urine sample without traces of opioids (morphine, methadone, buprenorphine, codeine, tramadol, ketobemidone, oxycodone, hydromorphone, dextromethorphan)	• Skin or nerve lesions or tattoos in the assessment areas
	• Regular use of analgesic drugs (≥ twice a week)
	• Allergy against morphine or other opioids (including naloxone)
	• Alcohol or drug abuse
	• Use of psychotropic drugs (except SSRI)
	• Neurological or psychiatric disease
	• Chronic pain condition
	• Use of prescription drugs 1 week before the experimental days
	• Use of over-the-counter drugs 48 hours before the experimental days

*ASA* American Society of Anesthesiology (physical status classification system), *BMI* body mass index, *NRS* numerical rating scale, *SSRI* selective serotonin reuptake inhibitors

The study protocol has been approved by the local Ethical Committee (*Videnskabsetisk Komité F for Region Hovedstaden*; H-15002712). The study will be conducted in accordance with the amended Helsinki Declaration.

### Setting

The experimental procedures of the study take place in a quiet examination laboratory (22–25 °C) at the Departments of Anesthesiology, 4231 and Neuroanesthesia, 3042 Rigshospitalet. Participants will adopt a comfortable supine position during the assessments.

### Study design

**Fig. 1:**
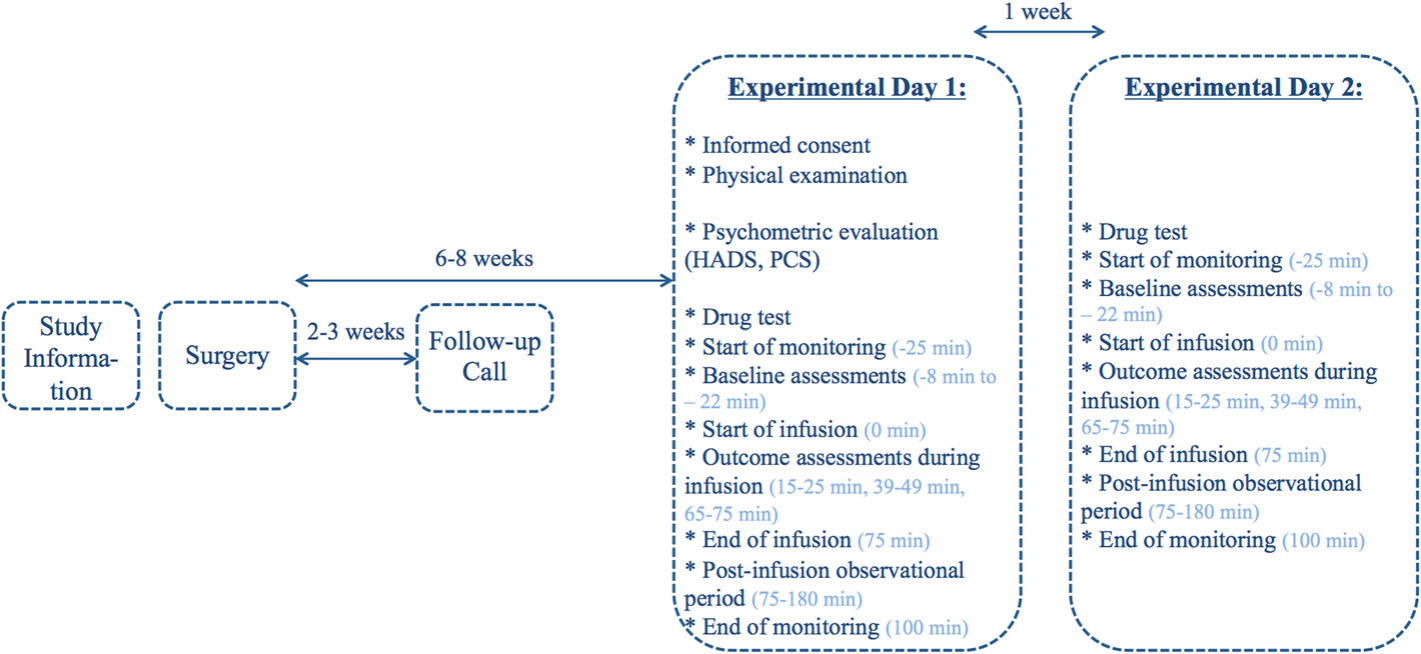
Study algorithm. The first experimental day (Day 1) will be performed 6–8 weeks following unilateral, primary, open groin hernia repair and will be separated by 7 days from the second experimental day (Day 2). Prior to the surgery, patients will receive oral and written information about the study. A follow-up phone call will be performed 2–3 weeks after surgery to assess the post-surgical pain level and eventual post-surgical complications. HADS, Hospital Anxiety and Depression Scale; PCS, Pain Catastrophizing Scale

This randomized, placebo-controlled, double-blind, crossover study consists of an information day prior to the surgery and 2 experimental days. A follow-up phone call will be performed 2–3 weeks after surgery to assess the post-surgical pain intensity and signs of post-surgical complications (swelling, erythema, wound-dehiscence, fever, malaise, excessive lymph node swelling). The study drug (naloxone or placebo) is given in a randomized fashion on the examination days, Day 1 and Day 2. Day 1 takes place 6–8 weeks after surgery and is separated by 7 days from Day 2 (Fig. 1).

**Fig. 2:**
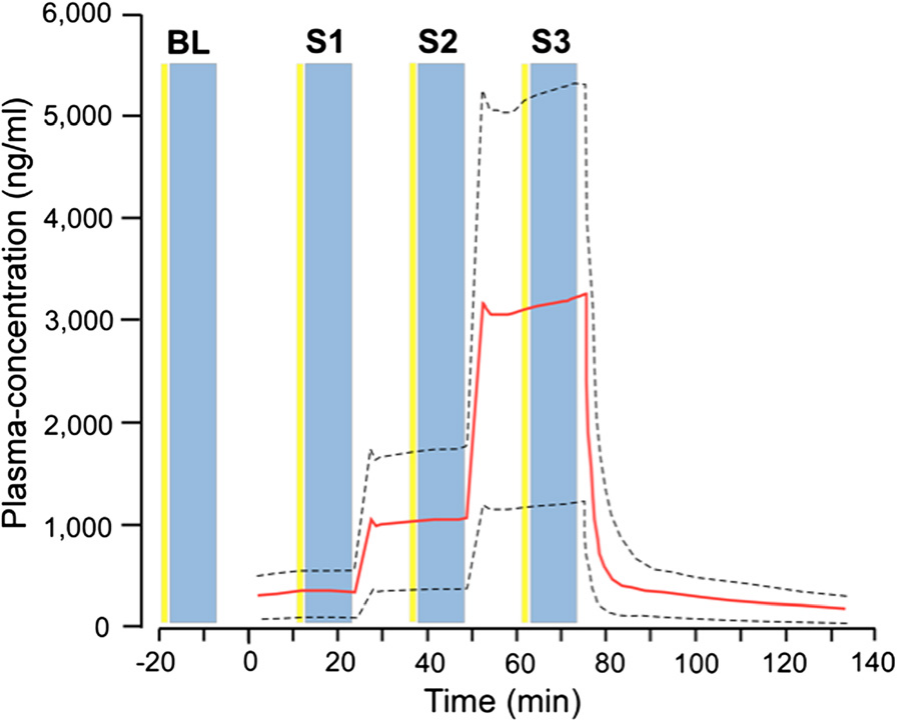
Day 1/2 test-algorithm with superimposed naloxone plasma-concentration curves. Mean plasma-concentration (red) with 95 % CI (dashed black lines) during a 3-step target-controlled infusion. Naloxone is given at Step 1 with 0.25 mg/kg, Step 2 0.75 mg/kg and Step 3 2.25 mg/kg. Yellow timeline columns represent ratings with Clinical Opiate Withdrawal Scale (COWS) and blue columns indicate sensory testing (pain during rest, movement and 100 kPa pressure; pressure algometry; secondary hyperalgesia area). BL, baseline assessments (−20 minutes to −8 minutes); S1, Step 1 assessments (15 minutes to 25 minutes); S2, Step 2 assessments (39 minutes to 49 minutes); S3, Step 3 assessments (65 minutes to 75 minutes)

On the information day, participants are orally informed by the investigator about the study and receive written information. On Day 1, participants are asked to sign an informed consent, if they meet the inclusion criteria. After a brief medical examination by a physician, participants are asked to fill out the Hospital Anxiety and Depression Scale (HADS) [23, 24] and the Pain Catastrophizing Scale (PCS) [25]. An instant urine drug screening test (morphine, methadone, buprenorphine, codeine, tramadol, ketobemidone, oxycodone, hydromorphone, dextromethorphan) is performed in order to avoid severe opioid-withdrawal reactions during the naloxone-administration. If a negative test result is obtained, baseline (−20 minutes to −8 minutes, Fig. 2) pain assessments, as well as assessments of secondary hyperalgesia and PPTs are carried out followed by administration of placebo or naloxone. Assessments will be repeated during infusion of placebo or naloxone at 3 different, estimated stable plasma concentrations (15 minutes to 25 minutes; 39 minutes to 49 minutes; 65 minutes to 75 minutes; cf. subsection below: Intervention; Fig. 2). On Day 2 experimental procedures are identical to Day 1.

### Randomization

Randomization will be performed by the Skanderborg Hospital Pharmacy using a randomization software (www.random.org). The pharmacy is also responsible for the packaging and labeling of naloxone (4 mg/ml) and placebo in identical 100-ml ampoules. Participants and investigators will be blinded to the administered infusions. Two copies of the randomization code for each participant are stored in a secure place, each in a sealed opaque envelope. One of the envelopes is placed in the Trial Master File of the study, while the other one is placed in the examination laboratory, to be opened only in case of a medical emergency.

### Intervention

Participants will be continuously monitored with electrocardiography, and, measurements of pulse oximetry, non-invasive blood pressure, heart rate and respiratory rate, beginning 25 minutes prior to the infusion (−25 minutes) and, if no adverse effects are observed, ending 25 minutes after termination of the infusion (100 minutes). Solutions of naloxone and placebo will be administered according to the randomization procedure and are identical in regard to color, density, transparency and odor.

The TCI-algorithm was calculated by the software NONMEM (7.3 ICON Development Solutions, Manchester, UK (property of University of California, San Francisco (UCSF), CA, USA)), using computer simulations based on a population-kinetic model with 2000 simulated administrations distributed on 10 subjects [26]. Thus, a total dose of 3.25 mg/kg of naloxone will be administered in a 3-step approach (Step 1: 0 minutes to 25 minutes; Step 2: 25 minutes to 50 minutes; Step 3: 50 minutes to 75 minutes, Fig. 2) as follows: in the first minute (0–1 minutes) a bolus of 0.02 mg/kg is given followed by an infusion of 0.23 mg/kg during 24 minutes (1–25 minutes). Afterwards (25–26 minutes) a bolus of 0.06 mg/kg is administered followed by a 24-minute infusion of 0.69 mg/kg (minutes 26–50). Finally, a bolus of 0.18 mg/kg (50–51 minutes) is given followed by a 24-minute infusion of 2.07 mg/kg (51–75 minutes; Table 2). A period of observation then follows (75 minutes to 180 minutes), after which participants are discharged. With this infusion-algorithm, estimated naloxone plasma concentrations (median (95 % confidence interval (CI))) of 344 ng/ml (130;567), 1059 ng/ml (400;1752) and 3196 ng/ml (1205;5276) at time intervals 15–25 minutes, 40–50 minutes and 65–75 minutes, respectively, are achieved (Fig. 2). An identical infusion-algorithm is used for the placebo infusion. All outcomes are assessed at baseline (−20 minutes to −8 minutes, Fig. 2) and during infusions (15 minutes to 25 minutes; 39 minutes to 49 minutes; 65 minutes to 75 minutes, Fig. 2).

**Table 2:** Naloxone (4 mg/ml) administered intravenously

	Time (min)	Dose (mg/kg)	Dose/70 kg (mg)	Volume/70 kg (ml)	ml/min/70 kg
Bolus 1	0–1	0.02	1.40	0.35	0.35
Infusion 1	1–25	0.23	16.10	4.03	0.17
Bolus 2	25–26	0.06	4.20	1.05	1.05
Infusion 2	26–50	0.69	48.30	12.08	0.50
Bolus 3	50–51	0.18	12.60	3.15	3.15
Infusion 3	51–75	2.07	144.90	36.23	1.51
TOTAL		3.25	227.50	56.88	

Timeline, dose/kg, dose/70 kg, volume/70 kg and infusion rates/min/70 kg for the 3 step target-controlledinfusion. The total naloxone-dose administered is 227.5 mg per 70 kg BW. The total naloxone volume is 56.9 ml per 70 kg BW. BW: body weight (kg)

Infusions will be discontinued, if participants’ pain ratings at rest ≥ 5 assessed by the numerical rating scale (NRS, 0–10). If necessary, analgesic given intravenously as rescue medication (alfentanil) will be given in a titrated fashion for acute pain relief. Other significant adverse effects that may occur will also be treated accordingly. Participants will be contacted by phone the day after each examination day to check up on potential side effects. All side effects will be recorded and reported to the Regional Committee of Research Ethics and the Danish Health and Medicines Authority.

### Primary outcome

Participants are asked to rate pain intensity (NRS) at baseline (−20 minutes to −8 minutes, Fig. 2) and during infusions (15 minutes to 25 minutes; 39 minutes to 49 minutes; 65 minutes to 75 minutes, Fig. 2) using 3 different standardized testing-conditions: (1) at rest in the supine position, (2) during transition from supine to standing position and (3) during a pressure stimulus (100 kPa) manually applied, perpendicularly at the skin at the point of maximum pain in the groin (superficial inguinal ring), using an electronic pressure algometer (Algometer, Somedic AB, Hörby, Sweden) with a 1-cm^2^ felt-tipped probe. The summated pain intensity (SPI) is calculated by adding the NRS scores recorded in the three conditions. The primary outcome is the difference in SPI (SPID) at baseline and after infusion of naloxone:

$$ \mathrm{S}\mathrm{P}\mathrm{I}{\mathrm{D}}_{\mathrm{Nx}} = \left[\mathrm{T}\mathrm{C}\mathrm{I}\hbox{-} \mathrm{S}\mathrm{P}{\mathrm{I}}_{\mathrm{Nx}}\right] - \left[\mathrm{B}\mathrm{L}\hbox{-} \mathrm{S}\mathrm{P}{\mathrm{I}}_{\mathrm{Nx}}\right],\ \mathrm{compared}\ \mathrm{t}\mathrm{o}\ \mathrm{placebo}: $$

$$ \mathrm{S}\mathrm{P}\mathrm{I}{\mathrm{D}}_{\mathrm{PL}} = \left[\mathrm{T}\mathrm{C}\mathrm{I}\hbox{-} \mathrm{S}\mathrm{P}{\mathrm{I}}_{\mathrm{PL}}\right] - \left[\mathrm{B}\mathrm{L}\hbox{-} \mathrm{S}\mathrm{P}{\mathrm{I}}_{\mathrm{PL}}\right], $$

(BL, baseline; Nx, naloxone, PL, placebo; TCI, during target-controlled infusion). The baseline SPI-score (summated NRS score) will be subtracted from the SPI-score (summated NRS score), recorded at the highest obtainable TCI step.

### Secondary outcomes

**Fig. 3:**
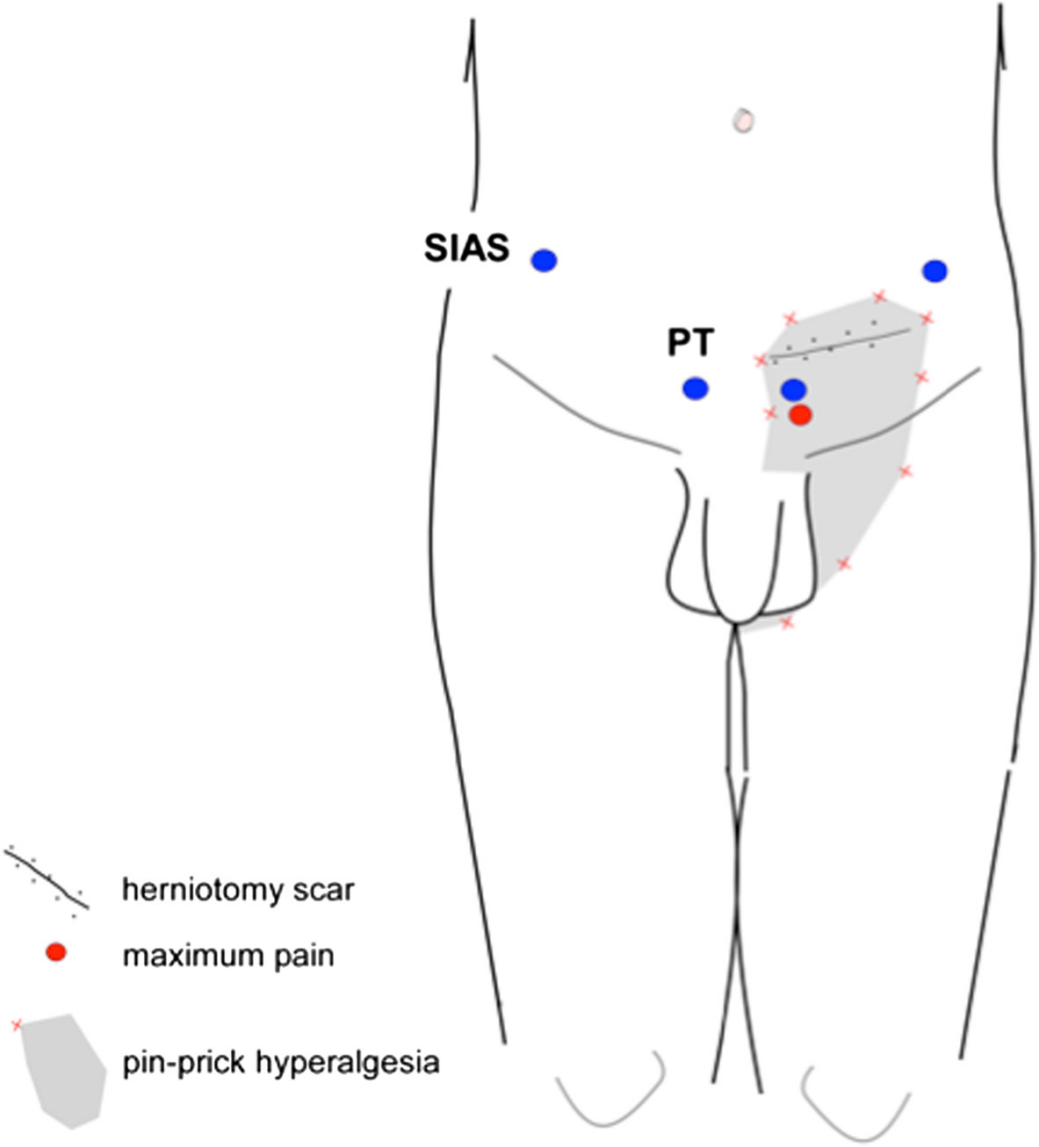
Examination areas. The examination areas exemplified with a subject after left-sided groin hernia repair. The shaded area, circumscribing the scar and delineated by sensory testing with a weighted-pin instrument (red crosses), represents a secondary hyperalgesia area. The maximum pain (red circle) is located above the left superficial inguinal ring. PT (blue circles), pubic tubercle; SIAS (blue circles), superior, inferior iliac spine

Assessments are performed at the surgical site and at the mirror-site in the contralateral groin (Fig. 3) at baseline (−20 minutes to −8 minutes, Fig. 2) and during infusions (15 minutes to 25 minutes; 39 minutes to 49 minutes; 65 minutes to 75 minutes; Fig. 2).

#### Secondary hyperalgesia/Allodynia

Secondary hyperalgesia and allodynia will be assessed using a weighted-pin instrument (MRC Systems, Heidelberg, Germany; 512 mN (10,424 kPa)) and a cotton wool bud, respectively. These assessments will be performed at baseline (−20 minutes to −8 minutes, Fig. 2) and during infusions (15 minutes to 25 minutes; 39 minutes to 49 minutes; 65 minutes to 75 minutes, Fig. 2) at the surgical site and at the mirror-site in the contralateral groin. The border of the hyperalgesic/allodynic area is determined by stimulating in 8 symmetric lines each separated by an angle of 45° converging towards the superficial inguinal ring. The stimulations start in normal skin outside the area of secondary hyperalgesia/allodynia and participants, with their eyes closed, are instructed to report a definite change in perception to an uncomfortable, burning or stinging sensation. The corners of the octagon are marked on the skin and transferred to a transparent, acetate sheet. The area is calculated using a vector-based drawing program (Canvas 12.0, ACD Systems International, Victoria, BC, Canada). Baseline outcome values of secondary hyperalgesia area/allodynia area will be subtracted from the scores of secondary hyperalgesia/allodynia area assessed at the highest obtainable TCI-step.

#### Pressure pain thresholds

PPT will be assessed using the electronic pressure algometer applied perpendicularly to the skin with successively increasing pressure at a rate of 1–2 kPa/s. PPT assessments will be performed at baseline (−20 minutes to −8 minutes, Fig. 2) and during infusions (15 minutes to 25 minutes; 39 minutes to 49 minutes; 65 minutes to 75 minutes, Fig. 2) at the surgical site and at the mirror-site in the contralateral groin. The assessments will include the point of maximum pain. Participants interrupt the stimulation by pushing a button when the pressure becomes painful. The cut-off value is 350 kPa. Values exceeding the cut-off are designated 351 kPa. Baseline outcome values of PPT will be subtracted from the scores of PPT assessed at the highest obtainable TCI-step.

#### Assessment of opioid withdrawal symptoms

Signs of opioid-withdrawal will be evaluated using the examiner-based Clinical Opiate Withdrawal Scale (COWS) [27] at the beginning of each experimental day and during infusions (13 minutes to 15 minutes; 37 minutes to 39 minutes; 63 minutes to 65 minutes, Fig. 2).

#### Psychometric evaluation

At the beginning of Day 1, participants are asked to fill out the HADS and the PCS for psychometric evaluation.

### Sample size calculation

The sample size calculation is based on the primary outcome: SPID before (−20 minutes to −8 minutes, Fig. 2) and during infusions (15 minutes to 25 minutes; 39 minutes to 49 minutes; 65 minutes to 75 minutes, Fig. 2) of naloxone:

$$ \mathrm{S}\mathrm{P}\mathrm{I}{\mathrm{D}}_{\mathrm{Nx}} = \left[\mathrm{T}\mathrm{C}\mathrm{I}\hbox{-} \mathrm{S}\mathrm{P}{\mathrm{I}}_{\mathrm{Nx}}\right]\ \hbox{--}\ \left[\mathrm{B}\mathrm{L}\hbox{-} \mathrm{S}\mathrm{P}{\mathrm{I}}_{\mathrm{Nx}}\right],\ \mathrm{compared}\ \mathrm{t}\mathrm{o}\ \mathrm{placebo}: $$

$$ \mathrm{S}\mathrm{P}\mathrm{I}{\mathrm{D}}_{\mathrm{PL}} = \left[\mathrm{T}\mathrm{C}\mathrm{I}\hbox{-} \mathrm{S}\mathrm{P}{\mathrm{I}}_{\mathrm{PL}}\right]\ \hbox{--}\ \left[\mathrm{B}\mathrm{L}\hbox{-} \mathrm{S}\mathrm{P}{\mathrm{I}}_{\mathrm{PL}}\right], $$

where TCI-SPI indicates the summated measure at the highest obtainable TCI-step. (BL, baseline; Nx, naloxone, PL, placebo; TCI, during target-controlled infusion).

The sample size calculation is based on a minimal relevant SPID-difference of 3 NRS-units, a significance level of 0.01 (*α*) and a power of 0.9 (*β* = 0.1). Since no past studies with similar design and hypothesis have been performed to support our assumptions, we opted for this calculation to use an assumed estimate of intra-individual standard deviation of mean BL-SPI of 1.2 NRS-units. Assuming normal data distribution and a crossover design, the estimated number of subjects needed is 9. We plan to include 16 subjects to compensate for eventual dropouts. If the assumed estimate of intra-individual standard variation is larger than expected: i.e., 1.7 or 2.5 NRS-units vis-à-vis 1.2 NRS-units, the corresponding sample size would increase from 9, to, 14 or 20, respectively (*P* < 0.01, power = 0.90).

### Statistics

To test if data are normally distributed, residual plots and the Kolmogorov-Smirnov test will be used (SPSS 20.0, Chicago, IL, USA, MedCalc Software: version 12.07.0.0; Mariakerke, Belgium). In case of non-normal distribution, primarily, logarithmic transformation is tried or secondarily, a Box-Cox transformation will be attempted for normalization of the data.

The primary outcome ΔSPID (SPID_NX_ − SPID_PL_) is analyzed by one-way repeated measures ANOVA (ΔSPID: dependent variable; TCI-steps: independent variable) or a corresponding non-parametric analogue (Friedman test), as appropriate. Paired *t* test or Wilcoxon rank sum test will be used, as appropriate, for inter-group comparisons. A mixed model with random effect for subject and fixed-effects for the factors: *target*-*controlled infusion* (Step 1/Step 2/Step 3), *secondary areas of hyperalgesia*, *PPT*, *HADS*-*scores* and *PCS*-*scores*, is used for the primary outcome ΔSPID (SPID_NX_ – SPID_PL_). Non-significant factors (*P* > 0.05), beginning with interactions, are excluded until all included factors attain significance. Main-effects and interaction-effects are examined.

The risk of type I error is reduced by setting a significance level of 0.01 (*α*). A power of 0.9 (*β* = 0.1) was chosen to reduce the risk of type II errors. For all statistical calculations, in which multiple comparisons are performed, Duncan’s new multiple range test or Scheffé’s method, as appropriate, will be used. Statistical calculations will be performed with partially un-blinded data: i.e., groups A and B. Parametric data will be given as mean (95 % CI), while non-parametric data will be presented as median (95 % CI).

### Study management

The study will be conducted in accordance with the guidelines and rules concerning quality control and quality management on clinical trials involving humans, and will follow the Good Clinical Practice and the Good Manufacturing Practice guidelines. All data about potential and enrolled participants will be treated confidentially. Only the investigators and relevant authorities will have access to the data. Eventual amendments to the protocol will be communicated to the relevant authorities and the entries on the registry databases will be updated. Positive negative or inconclusive results will be sought published. The study is registered in EUDRACT (2015-000793-36) and at the international database clinicaltrials.gov (NCT01992146).

## Discussion

Recent animal studies performed after resolution of inflammatory pain showed reinstatement of tactile hypersensitivity following administration of MOR-antagonists, suggesting latent sensitization mediated by endogenous opioids [6, 7], serving as a protective mechanism [15–17]. The aim of this study is to show, in humans, latent sensitization mediated by the endogenous opioid system in a clinical scenario.

Regarding the naloxone dose, a positron emission tomography (PET) study from 1989 indicated complete inhibition of [^11^C]-carfentanil binding to MOR following administration of 0.1 mg/kg naloxone [28] in human volunteers. The present authors are not aware of other studies replicating these data with newer technologies. However, a total dose of 3.25 mg/kg of naloxone, which is 32.5 times higher than the estimated MOR-blocking dose, and additionally, approximately 500 to 5000 times higher than the clinical dose used in the treatment of an opioid overdose [29], will be administered in this study. The reason for selecting the high dose of naloxone was twofold. First, by back-translating the human burn-injury model to rodents, following administration of high doses of naloxone (3.0 and 10.0 mg/kg), but not low doses (0.03 and 0.3 mg/kg), we observed signs of late reinstatement of hypersensitivity 21 days after an inflammatory injury [11]. Second, a low-dose naloxone infusion (0.021 mg/kg) [10] did not reinstate secondary hyperalgesia following resolution of a first-degree burn-injury, while a high dose (2 mg/kg) induced reinstatement in 4 out of 12 volunteers [11]. Thus, our translational data from rodents indicate that a high naloxone dose too is necessary to induce late reinstatement of hypersensitivity in humans. We attempt in the present study, to reinstate pain at three gradually increasing, but stable plasma concentration levels of naloxone, expecting in this way to obtain a pain reinstatement dose-response curve.

Concerning the risk of adverse effects induced by administration of a high-dose naloxone infusion, we expect to observe only mild and transient effects. In the translational study, 2 mg/kg of naloxone was administered to 3 pilot volunteers and 12 study volunteers. Six volunteers reported mild transient adverse effects, including tiredness, epigastralgia, frontal headache and photophobia. Furthermore, naloxone did not induce any overt changes in electrocardiography, blood pressure, heart and respiratory rate and oxygen saturation during the experimental days [11]. Additionally, doses up to 6 mg/kg have been used parenterally in previous studies both in healthy volunteers [18–22, 30] and in patients [31–36], and, only mild to moderate transient adverse effects have been recorded. We are, therefore, confident that the risk of development of complications or serious adverse reactions associated with the administration of a total naloxone dose of 3.25 mg/kg is highly unlikely. Moreover, in the present study we are using TCI to increase the naloxone plasma concentration step-wise, allowing blood-brain equilibrium to be obtained. Since the estimated steady-state conditions are obtained in less than 15 minutes [37], the adverse reactions can, therefore, be detected much earlier and at a lower naloxone dosage, compared with a single-bolus-based regimen.

In regard to the risk of development of a sustained pain state after the naloxone administration in patients after GHR, we consider it highly unlikely. Naloxone is a short-acting reversible antagonist of opioid receptors and does not affect the production of endogenous opioids. Moreover, a prolonged reversal of endogenous opioids is unlikely due to the pharmacokinetic properties of naloxone. In adults, the distribution half-life of naloxone is 40 to 70 seconds [38], and the elimination half-life, 54 to 64 minutes [38, 39] and no known, active long-acting metabolites are formed. Furthermore, in the previously mentioned rodent study [7], administration of naltrexone or naloxone led to transient episodes of hypersensitivity with duration of 60 to 90 minutes, while repeated administration of naltrexone, over the course of months, has confirmed full reversibility of the hypersensitivity episodes. In addition, our data from the high-dose naloxone (2 mg/kg) study in volunteers only indicated very short lasting changes in hypersensitivity [11].

The experimental days are taking place 6–8 weeks after surgery, time-points at which patients are nearly pain-free, albeit before the surgically induced tissue injury has fully resolved. Our primary outcome is a summated measure of reinstatement of pain (at rest, during transition from supine to standing position and evoked by pressure algometry), since pain is the most relevant outcome in the clinical setting. For the sample size calculation we opted to use a minimal relevant difference of 3 NRS-units, which is relatively low, considering our use of a summated measure (SPI) of 3 assessments (resting pain, movement-related pain and pressure evoked pain). Baseline SPI is estimated to be 8 (mean per assessment = 2.7) NRS-units: a significant increase would then be ≥ 11 (mean per assessment ≥ 3.7) NRS-units: i.e., ≥ 38 %.

Additionally, secondary outcomes (secondary hyperalgesia/allodynia and PPT), which are of exploratory nature, will provide information on sensory properties of the surgical site and of the mirror-site in the contralateral groin, and how they are modulated by naloxone.

Since secondary hyperalgesia is a measure of central sensitization, it is of interest to differentiate between subjects developing large secondary areas of hyperalgesia (high-sensitizers) and subjects developing small areas (low-sensitizers). Past research has shown that the size of the secondary area of hyperalgesia is reproducible within subjects and can thus be regarded as a phenotype [40]. It is unclear whether different sensitization phenotypes have varying susceptibility to develop latent sensitization. Interestingly, data from the translational study indicated that volunteers showing reinstatement of secondary hyperalgesia by naloxone administration exhibited significantly larger baseline areas of secondary hyperalgesia compared to naloxone non-responders. However, due to the small number of volunteers showing reinstatement (*n* = 4) cautious interpretation is required [11]. This is in agreement with the study by Corder et al., which used strain C57BL/6 rodents [7], known to have enhanced sensitivity to pain [41, 42]. In the present study, the magnitude of the secondary area of hyperalgesia at the surgical site will be correlated with the magnitude of pain reinstatement (SPID). This may thus provide important insights on whether a specific subgroup of subjects is more predisposed to latent sensitization.

In conclusion, by administrating a high-dose TCI of naloxone, the present study aims to show dose-dependent reinstatement of pain and hyperalgesia following recovery from a unilateral, primary, open GHR procedure. This would provide further evidence of opioid- mediated latent sensitization in humans. We consider that impairment of the protective endogenous opioid system may play a relevant role in the development of persistent post-surgical [43] or post-traumatic pain: thus, the long-term goal of alleviating persisting pain should be focused either at facilitating the endogenous opioid analgesia, restricting latent sensitization within a state of remission, or at measures that more effectively suppress latent sensitization [5].

## Trial status

Inclusion of the first participants is expected in fall 2015.

## Abbreviations

BL: baseline
CFA: Complete Freund’s adjuvant
CI: confidence interval
COWS: Clinical Opiate Withdrawal Scale
GHR: groin hernia repair
HADS: Hospital Anxiety and Depression Scale
MOR: mu-opioid-receptor
NMDA: *N*-methyl-*d*-aspartic acid
NRS: numerical rating scale
Nx: naloxone
PCS: Pain Catastrophizing Scale
PET: positron emission tomography
PL: placebo
PPT: pressure pain threshold
SPI: summated pain intensity
SPID: summated pain intensity difference
TCI: target-controlled infusion
UCSF: University of California, San Francisco
