# Erratum to: ‘Effect of a high-dose target-controlled naloxone infusion on pain and hyperalgesia in patients following groin hernia repair: study protocol for a randomized controlled trial’

**DOI:** 10.1186/s13063-016-1181-z

**Published:** 2016-01-20

**Authors:** Manuel Pedro Pereira, Mads Utke Werner, Joergen Berg Dahl

**Affiliations:** Department of Anaesthesiology, Centre of Head and Orthopaedics, Rigshospitalet, Copenhagen University Hospitals, Copenhagen, Denmark; Multidisciplinary Pain Center, Neuroscience Center, Rigshospitalet, Copenhagen University Hospitals, Copenhagen, Denmark

Unfortunately, the original version of this article [1] contained an error. The author names were displayed incorrectly, Pereira MP, Utke Werner M, Berg Dahl J. The should have been written as follows: Pereira MP, Werner MU and Dahl JB.
